# Social dilemma in the external immune system of the red flour beetle? It is a matter of time

**DOI:** 10.1002/ece3.3198

**Published:** 2017-07-26

**Authors:** Chaitanya S. Gokhale, Arne Traulsen, Gerrit Joop

**Affiliations:** ^1^ Department of Evolutionary Theory Max Planck Institute for Evolutionary Biology Plön Germany; ^2^ Institut für Insektenbiotechnologie University of Giessen Giessen Germany; ^3^ Evolutionary Ecology and Genetics University of Kiel Kiel Germany

**Keywords:** public goods, social evolution, *Tribolium castaneum*

## Abstract

Sociobiology has revolutionized our understanding of interactions between organisms. Interactions may present a social dilemma where the interests of individual actors do not align with those of the group as a whole. Viewed through a sociobiological lens, nearly all interactions can be described regarding their costs and benefits, and a number of them then resemble a social dilemma. Numerous experimental systems, from bacteria to mammals, have been proposed as models for studying such dilemmas. Here, we make use of the external immune system of the red flour beetle, *Tribolium castaneum*, to investigate how the experimental duration can affect whether the external secretion comprises a social dilemma or not. Some beetles (secretors) produce a costly quinone‐rich external secretion that inhibits microbial growth in the surrounding environment, providing the secretors with direct personal benefits. However, as the antimicrobial secretion acts in the environment of the beetle, it is potentially also advantageous to other beetles (nonsecretors), who avoid the cost of producing the secretion. We test experimentally if the secretion qualifies as a public good. We find that in the short term, costly quinone secretion can be interpreted as a public good presenting a social dilemma where the presence of secretors increases the fitness of the group. In the long run, the benefit to the group of having more secretors vanishes and becomes detrimental to the group. Therefore, in such seminatural environmental conditions, it turns out that qualifying a trait as social can be a matter of timing.

## 1 INTRODUCTION

A social dilemma, such as the “tragedy of the commons” (Gordon, 1954; Hardin, 1968), describes a situation where individuals have to prioritize between following a selfish action, which pays them individually in the short run, or an altruistic action such as maintaining a public resource, which will be beneficial for the group in the long run. In evolutionary biology, it is typically argued that natural selection will work against such altruistic actions unless a mechanism that promotes cooperation is in place (Hamilton, 1964; Nowak, 2006b).

Social dilemmas may occur under all kinds of social interactions. For example, whether or not one should get vaccinated (or have children vaccinated) against infectious diseases such as measles, mumps, and rubella is a case of a social dilemma (Bauch & Earn, 2004). As vaccination might not prevent infection in 100% of cases and is not free of risk or side effects (Wu, Fu, & Wang, 2011), the ideal situation is when everyone else would be vaccinated, as it prevents the spread of infectious diseases and allows an individual to avoid the risks and costs of individual vaccination. To understand the underlying dynamics of such dilemmas, numerous mathematical models as well as experiments in various systems have been conducted (Kerr, Riley, Feldman, & Bohannan, 2002; MacLean, Fuentes‐Hernandez, Greig, Hurst, & Gudelj, 2010; Maclean & Gudelj, 2006; Nahum, Harding, & Kerr, 2011; Nowak, 2006a; Sigmund, 2010; Sokoloff, 1972, 1974, 1977) some of which present resolutions to the dilemma either via ecological processes or by effectively changing the interactions.

Here, we use *Tribolium castaneum* as a model system for inquiring whether the interactions resulting in a social dilemma are in fact consistent over time or merely as a result of when the observations were made (McCauley & Wade, 1981). The red flour beetle is used in various biological disciplines, for example, developmental biology, immunology, evolutionary ecology (Joop, Roth, Schmid‐Hempel, & Kurtz, 2014; Kittelmann, Ulrich, Posnien, & Bucher, 2013; Richards et al., 2008; Roth et al., 2010; Zou et al., 2007) as a model invertebrate model organism. With short generation times of about 1 month, the beetles can be readily subjected to experimental evolution. Adult beetles have the innate immune defense, but also are capable of an external quinone‐rich secretion with broad antimicrobial activity (Prendeville & Stevens, 2002). This secretion has been termed as an external immune defense (Joop et al., 2014) or alternatively, extended immune defense (Joop & Vilcinskas, 2014; Otti, Tragust, & Feldhaar, 2014). Producing the external secretion is a genetically controlled trait and costly (Joop et al., 2014; Li et al., 2013) thus being a classic costly trait. This secretion can be toxic to the beetle larvae—and the secretors themselves—at high concentrations (Joop et al., 2014; Sokoloff, 1972). Flour beetles live in proximity to each other and typically can be found in grain stores or flour mills, as human commensals (Levinson & Levinson, 1994). As the antimicrobial secretion acts in the environment of the beetle, it is potentially also advantageous to other individuals living in their proximity, including their offspring (Masri & Cremer, 2014). However, it remains to be tested whether the beetles actively control the antimicrobial secretion and if it can be treated as public good, thus imposing a social dilemma in the beetles (Cotter & Kilner, 2010). If the beetles can indeed strategically modify their secretion of quinones to suit others and/or depending on environmental conditions, such as the presence of parasites or pathogens then this would provide conclusive evidence of the secretion being a social trait.

Due to above‐mentioned side effects of high concentrations of secretion, we hypothesize a population level upper threshold which if crossed, is potentially harmful to all offspring and the beetles themselves, resulting in reduced fitness. One would also expect a lower threshold, below which the sufficient spreading of quinones into the environment is not enough to provide antimicrobial property. Hence, beetles would need to specifically control the amount they secrete into the environment. As other individuals can utilize the secreted product, secretion may be viewed as a social trait. The dilemma would be to either contribute to this common good and paying the costs or not to contribute, saving the costs and benefiting from the secretion of others. In the latter case, secreting beetles could compensate for nonsecreting individuals in the population to secure an optimal quinone concentration in the environment.

In this study, we address if quinone production meets the requirements of being a social trait. If populations of secretors are more successful at proliferating than populations of nonsecretors and if in any mixed population, the nonsecretors perform better than secretors, then quinone secretion could be classified as a social trait: Secretion would increase the average fitness of the group at a direct cost to the providing individual. If there is no cost of producing and inhabiting a quinone‐rich environment, then the average fitness of a group increases with the number of secretors and all secretor groups do better than a group of nonsecretors. Here, we tackle these two questions experimentally.

## 2 METHODS AND EXPERIMENTAL DESIGN

*Tribolium castaneum* are convenient to breed in the laboratory, using a climate chamber (32°C, 70% humidity), and population size as well as environment can be readily manipulated, for example, by introducing parasites and pathogens. Overlapping generations are the natural scenario, and therefore, all life stages are affected by the external immune defense being present in the environment. Hence, the costs of external secretion, as well as its efficacy, should manifest in the beetles’ fitness. Ideally, one would measure such as lifetime reproductive success. However, as the beetles can survive for several years under laboratory conditions, we took population growth as a proxy for fitness.

The environmental presence of the external secretion indeed protects the (nonsecreting) beetle offspring from microsporidian parasitic infections within one generation of exposure (Joop et al., 2014). However, when exposed to the microsporidia over a longer time frame, the beetles went extinct (Rafaluk et al., 2015). To avoid colony collapses, here we used a fungus, *Beauveria bassiana*, which upon single exposure is affected by the external secretion of the beetles while in the long run becomes resistant and more virulent (Rafaluk et al., 2017). Here, we chose a fungus spore concentration of 10^8^ spores/g of flour for the preparation of the spore‐flour mixture (Rafaluk et al., 2017). As a stress control and to control for host–parasite interactions, we also introduced a treatment using heat‐killed spores of the fungus. Therefore, we had three levels of pathogen (none/control, noninfecting [heat killed] *B. bassiana* and infecting *B. bassiana*). We investigated whether

- On an individual level, secretors compensate for nonsecretors,
- The average fitness of the group increases with the number of secretors, and
- The fitness of populations with only secretors is greater than the fitness of the populations with no secretors.

**Figure 1:**
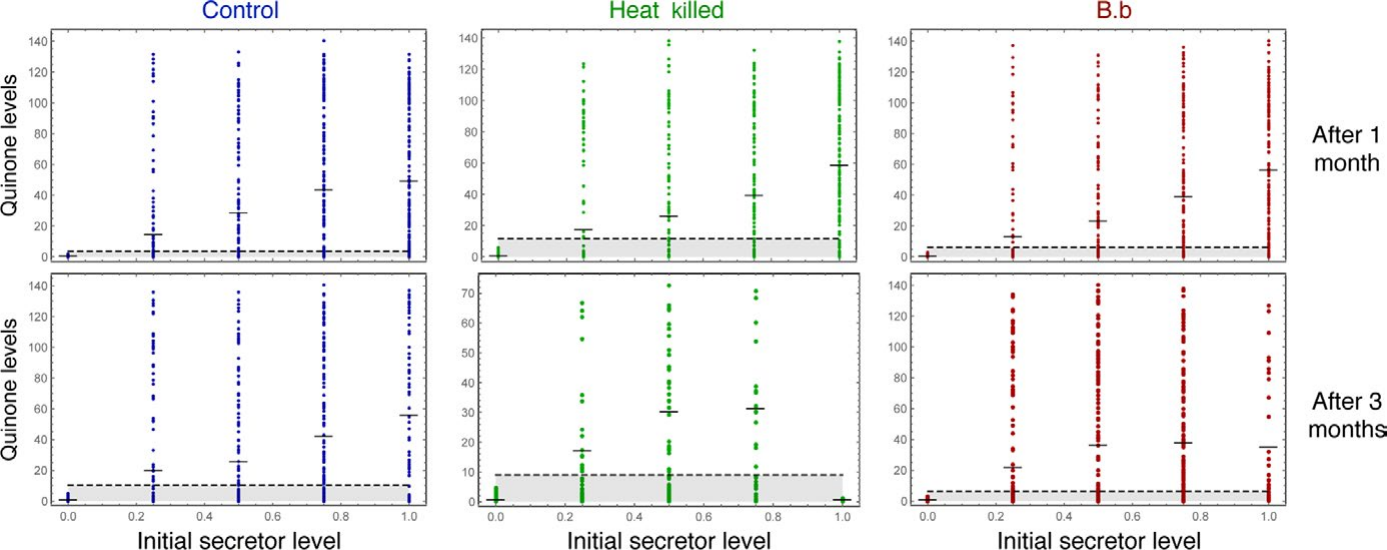
Defining secretors versus nonsecretors: The individual quinone levels where assessed for 30 randomly sampled individuals from each replicate are represented as a dot. In total, the aim was to get 150 individuals from each treatment. In some cases, the population size was not large enough for these measurements. Even in cases where there are no secretors, there is a basal level of expression. As a conservative assumption, we set a level twice that of the maximum basal expression (given by the horizontal dashed line) to differentiate between secretors and nonsecretors. We then extrapolate this measure to the population sizes given in Figure 3

To address these topics, we first needed to define secretors versus nonsecretors at an individual level because the beetles produce different levels of quinones. Within the “nonsecretors”, individuals produce very low levels of quinones, but there are none that do not produce any. For each treatment, we had one population, which was composed of all low‐level secretors. The level of quinone secretion which this low‐level secretor population reached in each particular treatment was taken to be the baseline quinone secretion for that treatment (Figure 1). Quinones of interest auto‐fluoresce with a peak at 245 and 246 nm. To fully capture the quinone secretion on a plate reader, we took measures from 210 to 262 nm and calculated the area under curve (Joop et al., 2014; Rafaluk et al., 2017). As a conservative estimate, all the individuals secreting more quinone than twice the baseline secretion were considered to be secretors, for that treatment.

To address the point of compensation for the presence of nonsecretors, we first needed to establish a baseline of the noncompensatory scenario. From the parental population which was used to seed the different replicates, the mean level of quinone secretion of the secretors was *q*_s_ = 57.74 (*n* = 46) (measured as above) and that of the nonsecretors was *q*_ns_ = 1.82 (*n* = 46). We normalized these means such that they formed the end points of a continuum from 0 to 1 ((1.82 − *q*_ns_)/(*q*_s_ − *q*_ns_) to (57.74 − *q*_ns_)/(*q*_s_ − *q*_ns_)). Thus, a group with only secretors coming from this population should theoretically produce quinones at level 1 and conversely all nonsecretor groups at level 0. Additionally assuming a linear accumulation of quinones, a group made up of *i* secretors, and *N* − *i* nonsecretors should produce *i*/*N* level of quinones (again scaled between 0 and 1).

We manipulated the initial ratio of secretors to nonsecretors in a population with a given population density and carrying capacity. Here, we used one beetle per 0.1 g flour, 60 beetles per population in 6 g flour (Blaser & Schmid‐Hempel, 2005). The beetle lines originated from an evolution experiment on quinone secretion, selecting for either high (=secretors) or low (=nonsecretors) quinone secretion (Joop et al., 2014). We implemented five ratios of secretors: nonsecretors (100% secretors, 75:25, 50:50, 25:75, 100% nonsecretors), each level being replicated five times. Thus, the experiment was performed as a fully factorial setup, resulting in a total of 75 populations per time point, with the time points being 1 and 3 months.

As the beetles influence their environment due to their external immune secretion as well as other excretions, the environment is not constant in our experiment but changes over time. The secreted quinones spread, oxidize to hydroquinones which also have antimicrobial property, and persist. Therefore, populations started in quinone‐free environment (with or without pathogens, depending on the treatment), while later generations as well as aging adults experienced a quinone‐rich environment. The environment got enriched with additional excretions and most likely contained less food or food of lower quality (compare Joop et al., 2014 for quinone flux into the environment). As mentioned, *T. castaneum* can live under laboratory conditions for several years. In the wild, however, lifetime is expected to be shorter. To consider the changing environment, we measured the population growth at two time points, after 1 month and after 3 months, representing a likely natural life span of an adult and allowing for about three generations in the 3‐month treatment including the starting generation. The groups that were set up had been randomly assigned to a 1‐month or 3‐month treatment. This approach avoided the problem of disturbing the *Tribolium* jars after a month (including the danger of eggs/larval wounding during sieving, changing the flour structure).

At the end of the assay, that is, after 1 or 3 months, all individuals per jar were counted and separated by life stage. Besides, quinone secretion was measured for thirty randomly selected individuals to calculate the mean quinone level (compare Joop et al., 2014). In cases where less than thirty adults were present in the population, all remaining adults were measured.

## 3 RESULTS

### 3.1 Secretors compensating for nonsecretors

**Figure 2:**
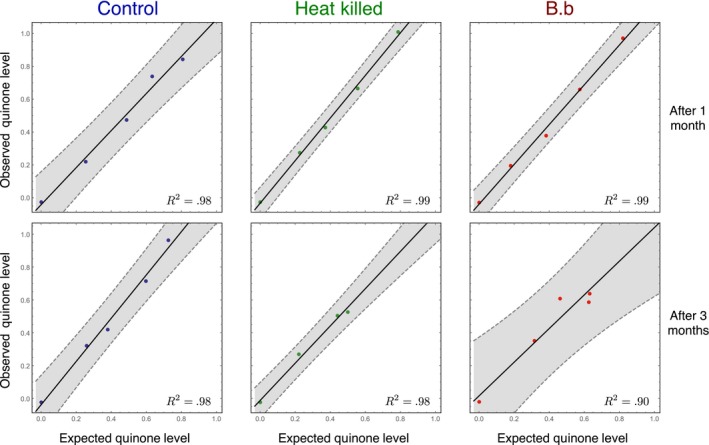
Comparing the observed quinone levels from the experiment with the expected levels calculated under linearity. If the secretor do not compensate for the nonsecreting beetles, then the amount of quinone expected in the populations would be linear as to the fraction of secretors in each population. The quinone levels lie between 0 and 1 as forming the ends of a continuum where 0 corresponds to *q*_ns_ = 1.82, the mean quinone level of a nonsecreting population and 1 to *q*_s_ = 57.75, the mean quinone level of a secreting population as from the seeding parental generation (see Section Results). Note that the expected quinone levels (fraction of secretors) are not constant as (0, 0.25, 0.5, 0.75, 1) but change with the fraction of secretors after 1 and 3 months as a result of the population dynamics (Figure 4). The expectation is based on the assumption that the production of quinones is unaffected or cannot be actively modulated by the secretor in response to the presence/absence of nonsecretors. The strong linear relationship between the shows that the secretors do not compensate/modulate their secretion for the presence of nonproducers. The solid lines are a linear fit to the data with a 95% confidence region shaded around it

We indirectly tested if secretors compensate for nonsecretors by comparing the quinone level after the experiment to the theoretical quinone level expected if the beetles did not compensate for nonsecretors. For example, the 75:25 secretors: nonsecretors populations should have about 75% of the quinone secretion of an all secretor population within the given treatment. We could come to the conclusion that secretors compensate for nonsecretors if the three different secretors: nonsecretors populations would show significantly higher quinone secretion than expected from this calculation. To assess the increase/decrease in the level of quinones secreted by the beetles, first we established a baseline which could be compared to the theoretically expected value as defined in the Methods section. The actual amount of quinone secreted in each cohort of different fractions of secretors was measured and again rescaled as per the Methods to fall between 0 and 1 defined by the parental generation. The data points in Figure 2 are not at the initial fraction of secretors as we take into account the correction due to the changed fraction of secretors over time. We find minimal deviation from the expected amount of quinone (Figure 2). The secretors did not over (or under) compensate for the presence (absence) of nonsecretors. Thus, the individual quinone production does not seem to be under any active control by the beetle itself.

### 3.2 Population growth

**Figure 3:**
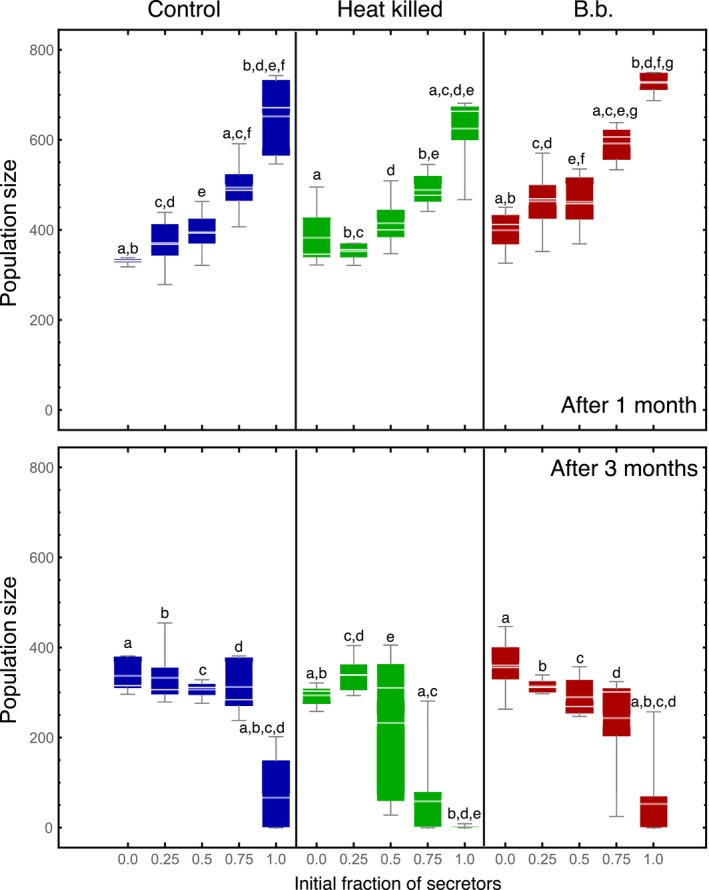
Population size dynamics in the three treatments for the different initial ratios of secretors: nonsecretors. In the first month, the all‐secretors group achieves the largest population size in all three treatments, with the growth being monotonic in the number of secretors at the initial conditions. After 3 months, however, in all three treatments, the all secretors suffer population crashes. This could be due to the population reaching its ecological limit the earliest as compared to others due to the initially fast growth. Also, it is possible that the amount of quinone produced cannot be controlled individually by the beetles and thus a population crash results due to excessive quinones becoming toxic. Within each treatment (and control) panel, we performed an ANOVA between the distributions. The final increase (after 1 month) or decrease (after 3 months) is always significant. For the control, heat‐killed and *Beauveria bassiana* treatments after 1 month, the results were significant at *p* < 0.05 level *F*_4,20_ = 22.39, *p* = 3.7 × 10^−7^, *F*_4,20_ = 22.39, *p* = 4.8 × 10^−6^ and *F*_4,20_ = 22.39, *p* = 5 × 10^−8^, respectively, indicated by like letters. After 3 months, the decrease in population size is significant again with *F*_4,20_ = 16.71, *p* = 3.6 × 10^−6^, *F*_4,20_ = 11.49, *p* = 5.2 × 10^−5^ and *F*_4,20_ = 10.04, *p* = 1.2 × 10^−4^ for control, heat‐killed and *B. bassiana* treatments, respectively

**Figure 4:**
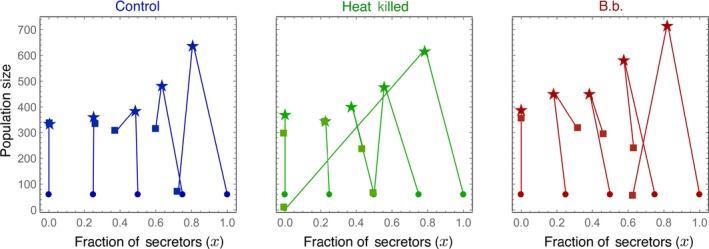
Population dynamics is usually excluded from the analysis of evolutionary dynamics. The experiment begins at the points indicated by circles, the 1‐month time point represented by stars, and the 3‐month time point is given by squares. However, when we look at ecological and evolutionary dynamics together we see that all treatments experience, a growth in population size, which reaches around 300 individuals in almost all populations except with all secretors (and the one with 75% secretors in the heat‐killed treatment) before reducing. This might indicate that for the given environment, a population limit is reached with about 300 individuals. Furthermore, the more initial secretors the stronger the drop down, potentially indicating that the upper quinone‐threshold was crossed. This in combination with expending the environment finally results in extinction, whereas populations which start with less secretors grow slower and thus still survive after 3 months

We take the growth rates of the populations as proxies for fitness. If the secretion of quinones is a public good, then the fitness of the group would increase with the number of secretors in it. Thus, the all secretor groups would have the highest fitness—population size—of all. After 1 month, the fitness of the populations increased with the fraction of secretors increases, in all three treatments (Figure 3, 1 month). Within most of the mixed populations, the fraction of secretors decreases over time; that is, they generally do worse than nonsecretors (Figure 4). Thus, this points to the existence of a social dilemma based on the expression of quinones. Note that in contrast to the general trend, the population trajectories starting at 0.5 in *B. bassiana* and heat‐killed treatments and at 0.25 in all three treatments show either minimal deviation from the starting fraction (Figure 4).

If we instead focus on the results after 3 months, the interpretation is very different (Figure 3, 3 months and Figure 4). After 3 months, the fitness of the population decreases with the initial fraction of secretors, implying no social dilemma between secretors and nonsecretors.

### 3.3 Population dynamics

An important difference between different time points is the population dynamics: Population size increased rapidly over the first month, but after 3 months, the population had declined substantially again (Figures 3 and 4). It seems that effects of the selection procedure and stochasticity become more pronounced only later (Figure 4). All populations decreased in size after 3 months. However, the fraction of secretors at both time points, in most of the treatments, decreased, indicating that nonsecretors are advantageous. This again hints at a social dilemma, but the slowed‐down growth of populations with more secretors after 3 months clearly shows that one cannot speak of a social dilemma in this system in general.

As for the population sizes, specifically, after 3 months with the different initial conditions, we found the following:

- 0% secretors—By definition, there are no secretors in all three treatments for this initial condition. For *B. bassiana* and the control treatment, the final populations were around 350, while for the heat‐killed version, they were slightly lower around 300.
- 25% secretors—The final population size in all three treatments stabilized at around 300. The fraction of secretors stabilized to around 20%–25% for the control and the heat‐killed treatments but with active pathogen pressure in the *B. bassiana* treatment, it increased to about 35%.
- 50% secretors—For the *B. bassiana* and the control, the final population sizes were close to 300. The final population size was slightly lower for the heat‐killed treatment. The final secretor levels for the two pathogen treatments stayed almost close to 50%, while for the control, it dropped to 40%.
- 75% secretors—We observed a major population crash in the heat‐killed treatment with the fraction of secretors close to 50%. This was followed by the *B. bassiana* treatment which reached a population size of 250 with 65% of secretors, while the control had a higher population size of 300 also with about 60% of secretors.
- 100% secretors—The heat‐killed treatment again suffered from the most drastic population crash with the fraction of secretors along‐with. For the *B. bassiana* treatment, the population also decreased in size, but the fraction of secretors remained substantial at 60%, while in the control, the fraction of secretors was close to 70% albeit in a small population.

While in the short term (1 month), all‐producer populations did best, in the long term (3 months) mixed populations with <50% secretors appeared to be more stable (Figures 3 and 4). This could arise from the limited environment, where quinone levels aggregate beyond the optimal level. Most importantly, this again proposes a fixed rather than a flexible strategy in quinone secretion. Seemingly, individuals are not adapting their secretion to environmental parameters such as pathogens or population composition or environmental quinone concentration, but the ratio of secretors to nonsecretors may be underlying the maximal group benefit. This is also reflected in the change in the population sizes, wherein the initially all‐producer cases growth rate drops to almost zero after an initial boost (Figure 3).

## 4 DISCUSSION

Our study shows that it is not possible to conclude that an observed “social” trait presents a social dilemma in general. It might very well be possible that the trait that seems social might be a simple by‐product of a natural phenomena one which cannot be controlled. In case of the external immune system of *Tribolium*, are other beetles getting a benefit from the producer beetles just as a by‐product? In that case, it would not be a social trait. This depends on the cost of producing the antimicrobial products. While it has been previously shown that indeed it is costly to produce quinones (Joop et al., 2014; Li et al., 2013), this does not mean that it would be necessarily amenable to manipulation by the secreting beetles themselves. Herein, we show that population density, environmental concentration, pathogen presence, and the proportion of secretors versus nonsecretors do not trigger an active regulation of quinone production in *T. castaneum*.

We show that in a very simple setup, a social dilemma can be found after 1 month but if we look at the all the results together and not just only population dynamics or only trait dynamics, then after 3 months, there is no sign of a social dilemma. Thus, the time point at which experiments are studied can be crucial in determining whether a social dilemma is present or not. Extrapolating an observation as a life‐history trait can result to misleading conclusions about experimental and empirical systems. Recently, for example *Pseudomonas* bacteria came under scrutiny for similar reasons. Interpreting any external secretion as a cooperative trait is misleading where every interaction could then be cast in the sociobiological framework (Foster, Parkinson, & Thompson, 2007; Nadell, Xavier, & Foster, 2009; Rainey, Desprat, Driscoll, & Zhang, 2014; West, Griffin, Gardner, & Diggle, 2006). An external secretion can be interpreted as a public good in a certain case while not in others and this can depend on the genotype–environment interaction (Rainey et al., 2014; Zhang & Rainey, 2013). Thus, where the natural environment of an organism is not provided, any desired result could potentially be obtained by manipulating the environment. Yeast is another example where the environment can easily produce an interaction pattern capable of being captured by a social dilemma of one form or the other: Invertase produced by some yeast cells hydrolyses sucrose into glucose and fructose which are then available for uptake. However, in natural populations, not all yeast cells produce invertase. Thus, avoiding the invertase production costs, the nonsecretors can uptake the simpler sugars owing to the presence of the secretor yeast cells. While it is tempting to classify this system as an example of a social dilemma, over time as new data have emerged, the perceptions have changed (Gore, Youk, & van Oudenaarden, 2009; Greig & Travisano, 2004; MacLean et al., 2010). What was thought of as a tragedy of the commons was then interpreted as a snowdrift game just to be later demonstrated as not to be a social dilemma at all.

In a similar fashion, here we show that it is possible to interpret the *Tribolium* system as appropriate to study social evolution (in particular, social dilemmas) or not, depending on the duration of the study. The potential common good in this system would be the quinone secretion with its antimicrobial property, being produced by adults only but with potential benefit to all life stages. Furthermore, *Tribolium* beetles are capable of mate choice (Peuß, Eggert, Armitage, & Kurtz, 2015) as well as of kin‐recognition (Jasienski, Korzeniak, & Lomnicki, 1988; Parsons, Zhong, & Rudolf, 2013). Should it be the case indeed that there are no metabolic costs, rather than we have not found them so far (thus resulting in cheating), an alternative explanation may be provided by actively regulating the population composition, for example, by cannibalism or differential dispersal rates between secretors and nonsecretors. Under environmental conditions requiring less external protection, such as a parasite‐free environment, there would be no need to take on the toxicity of quinone secretion and nonproducers might cannibalise producer–offspring, while in a contaminated environment, they would not do so to ensure the sufficient quinone supply for their offspring. So “cheating” would be shifted to a different level, which remains to be shown.

However, *Tribolium* species are not the only insects to secrete quinones. Bombardier beetles use it as predation defense, spraying it toward the predator to fend it off (Eisner, Jones, & Aneshansley, 1977), an interesting trait, in a solitary beetle. On the other hand, earwigs, subsocial insects providing facultative maternal care (Wong & Kölliker, 2012) and showing sibling cooperation (Falk, Wong, Kölliker, & Meunier, 2014) can produce and secret quinones in the larval as well as the adult life stage (Gasch, Schott, Wehrenfennig, Düring, & Vilcinskas, 2013; Gasch & Vilcinskas, 2014). For this species, quinone secretion has been discussed as predation defense in larvae (Gasch & Vilcinskas, 2014), while adults also use it when applying parental care to their eggs (Meunier & Kölliker, 2012). These examples point toward the actual ability of quinone production being rather evolutionary conserved, while its utilization seems to be diverse. Future research therefore not only should address the evolution of sociality as such, but considering it to be a gradient sensu lato (Cotter & Kilner, 2010).

Furthermore, our results reflect on how well experimental conditions represent the natural scenario. In the case of pathogen‐free conditions, it is possible that the secretion decreases to extremely low levels or might even be lost. We rely on the pathogen condition to test our hypothesis and do not consider long term dynamics, which should help to alleviate this potential issue (Andreas Mitschke, Momir Futo, Joachim Kurtz, Tina Gasch, Rayko Halitschke, Andreas Vilcinskas, Philip Rosenstiel, Hinrich Schulenburg and Gerrit Joop, unpublished work; Joop et al., 2014). Competition for food is another likely scenario persisting under various conditions, staying in a toxic environment appears to be less likely at least when considering motile organisms. For these, the solution to both problems would be dispersal, which is not permitted in most experimental setups. Thus, systems with time‐dependent conditions put us at risk of generating results fitting a theoretical expectation rather than objectively studying a natural system in an experiment. This stresses the importance of designing experiments which are informed but not constrained by the current theoretical thought before making final conclusions about the social interactions in the system.

## CONFLICT OF INTEREST

The authors declare no conflict of interest.
